# Drug Optimization in Patients with Mild-to-Moderate Ulcerative Colitis: A Global Survey

**DOI:** 10.3390/jcm13092510

**Published:** 2024-04-24

**Authors:** Ferdinando D’Amico, Vipul Jairath, Kristine Paridaens, Laurent Peyrin-Biroulet, Silvio Danese

**Affiliations:** 1 Department of Gastroenterology and Endoscopy, IRCCS Ospedale San Raffaele and Vita-Salute San Raffaele University, 20132 Milan, Italy; damico_ferdinando@libero.it; 2 Division of Gastroenterology, Department of Medicine, Western University, London, CA 91766, Canada; vipul.jairath@alimentiv.com; 3 Ferring International Center S.A., 1162 Saint-Prex, Switzerland; kristine.paridaens@ferring.com; 4 Department of Gastroenterology, Nancy University Hospital, F-54500 Vandœuvre-lès-Nancy, France; peyrinbiroulet@gmail.com; 5 Inserm, NGERE, University of Lorraine, F-54000 Nancy, France; 6 INFINY Institute, Nancy University Hospital, F-54500 Vandœuvre-lès-Nancy, France; 7 FHU-CURE, Nancy University Hospital, F-54500 Vandœuvre-lès-Nancy, France; 8 Groupe Hospitalier Privé Ambroise Paré—Hartmann, Paris IBD Center, 92200 Neuilly sur Seine, France; 9 Division of Gastroenterology and Hepatology, McGill University Health Centre, Montreal, QC H4A 3J1, Canada

**Keywords:** ulcerative colitis, inflammatory bowel disease, 5-ASA, budesonide MMX, optimization

## Abstract

**Background/Objectives**: The treatment of patients with mild-to-moderate ulcerative colitis (UC) is challenging. Although there are commonly used guidelines, therapy optimization is not standardized. We conducted a survey to investigate the management and treatment of patients with mild-to-moderate UC. **Methods**: Physicians with experience in treating inflammatory bowel diseases (IBD) were invited to participate in an anonymous, multiple-choice survey between June and July 2023. The survey addressed various issues of patient care such as patient monitoring, treatment optimization, follow-up, treatment decision making, and therapy de-escalation. **Results**: The survey included 222 physicians (59.9% men; mean age = 50.4 years) from 66 countries worldwide. Gastroenterologists were the most represented specialists (89.6%), followed by surgeons (3.2%), and internal medicine doctors (2.7%). Two-thirds of the participants (66.7%) had >10 years of experience in the field of IBD. The combination of oral (≥4 g/day) and rectal 5-aminosalicylic acid (5-ASA) was the preferred choice when optimizing therapy. Budesonide MMX (41.8%) and systemic steroids (39.9%) were preferred in patients who failed 5-ASA. Treatment decisions were predominantly based on endoscopic (99.0%) or clinical (59.8%) activity. A significant percentage of clinicians did not optimize therapy in the case of increased fecal calprotectin alone (45.1%) or radiological/ultrasound activity (39.8%) alone. **Conclusions**: The guidelines for the management of mild-to-moderate UC are well accepted in clinical practice. Endoscopic remission remains the main therapeutic target, followed by clinical remission. Fecal calprotectin and intestinal ultrasound still elicit complaints from physicians.

## 1. Introduction

Ulcerative colitis (UC) is a chronic inflammatory bowel disease (IBD) that negatively impacts patients’ quality of life [1]. Unlike Crohn’s disease (CD), UC was considered a non-progressive disease for years and surgery was recognized as a curative approach [2]. To date, growing evidence shows that patients with long-term UC experience a tubular colon with the loss of haustra, the narrowing, shortening, and stiffening of the colonic wall, fibrosis, and occasionally strictures [3,4,5]. Up to 30% of patients have an extension of their disease compared to the baseline over the course of ten years [6]. In addition, a considerable proportion of patients undergoing surgery require UC-related treatment, thus supporting the hypothesis that UC is a progressive disease [7]. This concept assumes that there is a ‘window of opportunity’ in the early stages of UC in which to intervene before inflammation becomes established [8,9]. For this reason, adequate monitoring and early treatment have a key role in the management of UC patients to avoid complications and improve disease control [10]. Although there are updated guidelines for the management of patients with mild-to-moderate UC, achieving disease remission is still challenging [11,12,13]. Several treatment options are available, including oral and rectal formulations of 5-aminosalicylic acid (5-ASA) and steroids. However, up to 70% of patients treated with 5-ASA do not maintain disease remission after one year of treatment [14]. Several factors affect the achievement of remission, including the change in therapeutic targets, which are no longer limited to the remission of symptoms but also include aims to achieve biochemical [normalization of C-reactive protein (CRP) and fecal calprotectin (FC)] and endoscopic remission [15]. Moreover, there are still non-standardized issues such as therapy optimization, the timing of medical therapy escalation, patient follow-up, and therapeutic de-escalation. We conducted a global survey to investigate the management and treatment of patients with mild-to-moderate UC, focusing on therapy optimization and patient follow-up.

## 2. Materials and Methods

A survey was designed to gather information from clinicians worldwide who are involved in IBD care. The survey was conducted from June to July 2023 using an online platform. The study was conducted and reported in compliance with the Consensus for Reporting of Survey Studies (CROSS) guidelines [16]. A CROSS checklist is available as Supplementary Materials. Survey invitations were distributed via multiple channels, including mailing lists of an IBD scope, a webinar platform for healthcare professionals interested in IBD, and personal invitations to physicians with an IBD focus [17]. The survey included screening questions at the beginning to ensure that respondents were part of the target population. Physicians not involved in the management of IBD patients were excluded. Email registration was used to prevent duplicate responses. Responses were collected anonymously. Permission for data collection was obtained from participants at the start of the survey. Both the survey and the invitation emails were in English. All questions in the survey were multiple choice. The questionnaire consisted of 52 questions grouped into six sections. The first section focused on demographics, specialty, and level of experience. The other sections covered various aspects of UC care, including the monitoring of patients in clinical remission, treatment optimization (e.g., 5-ASA ≥ 4 g per day or addition of rectal 5-ASA or steroids), follow-up after optimization, treatment decision making, and therapy de-escalation. The number of respondents for each question was reported to account for missing data, ensuring transparency in reporting. This study was conducted in accordance with the principles of the Declaration of Helsinki. The survey was non-interventional and was not intended to provide clinical data for treatment decisions; ethics approval was therefore not required. Informed consent was also not necessary as all data were completely anonymized [18]. 

## 3. Results

### 3.1. Demographics

In total, 222 physicians from 66 countries worldwide participated in the survey. The most represented countries were Italy (30/222, 13.5%), Brazil (15, 6.8%), Greece (14, 6.3%) and Israel (10, 4.5%) (Supplementary Materials). The mean age was 50.4 ± 11.0 years, and most participants were men (133, 59.9%). Gastroenterologists were the most represented specialists (199, 89.6%), followed by surgeons (7, 3.2%), internal medicine doctors (6, 2.7%), and other health care professionals (10, 4.5%). Most respondents had more than 10 years (148, 66.7%) or 5–9 years (46, 20.7%) of experience in the field of IBD. The number of IBD patients seen per year ranged from <100 (76, 34.2%), to <500 (77, 34.7%), and to <1000 (39, 17.6%). Only a small percentage of physicians visited >1000 patients per year (30, 13.5%). 5-ASA (160/163, 98.2%), systemic steroids (156, 95.7%), immunosuppressants (154, 94.5%), and biological drugs (152, 93.3%) were available to most physicians. Approximately three-quarters of participants had budesonide MMX (127/163, 77.9%) and small molecules (123, 75.5%) in their therapeutic armamentarium.

### 3.2. Monitoring of Patients in Clinical Remission

About half of the participants monitored their patients with mild-to-moderate UC after clinical remission <6 months (113/222, 50.9%), while, in the remaining cases, monitoring was performed <9 months (36, 16.2%), <12 months (32, 14.4%), <3 months (27, 12.2%), or >12 months (14, 6.3%) (Table 1). Remote visits through telemedicine were performed in about half of the cases (107/222, 48.2%), while only a small proportion of respondents (68/222, 30.6%) recommended the use of apps to monitor the clinical disease activity. A fecal calprotectin measurement was required <6 months in most cases (107/222, 48.2%), followed by assessments <12 months (42, 18.9%), <9 months (35, 15.7%) <3 months (19, 8.6%), and >12 months (19, 8.6%). Interestingly, the home testing of fecal calprotectin was used by only a fifth of physicians (45/222, 20.3%). Similarly, CRP was evaluated most frequently <6 months (77/152, 50.7%) or <3 months (30, 19.7%). Ultrasound and radiological examinations were requested by only half of the physicians (78/145, 53.8%), with a preferred frequency of every 6 (29/78, 37.2%) or 12 months (21, 26.9%). Colonoscopies/rectosigmoidoscopies were requested by about half of the respondents according to ECCO guidelines for colorectal cancer surveillance (100/197, 50.8%). However, there were physicians who requested endoscopic procedures once a year (32/197, 16.2%) or once every 2 years (43, 21.8%). Biopsies to evaluate histological activity were routinely taken in the majority of cases (117/145, 80.7%).

### 3.3. Treatment Optimization

**Figure 1:**
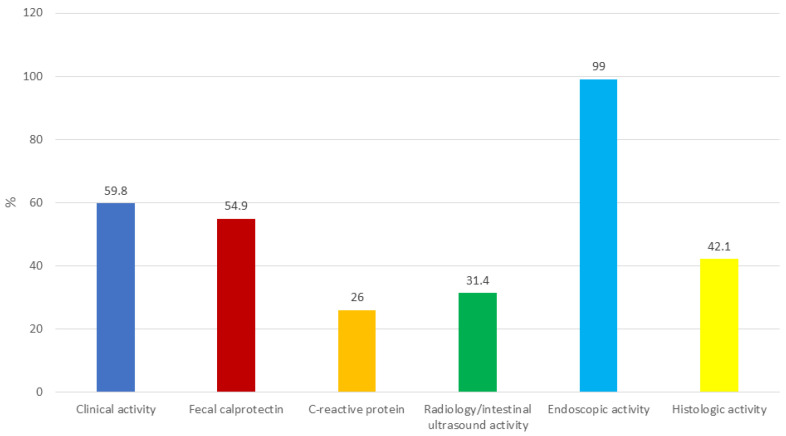
Treatment optimization stratified across different outcomes.

In most cases (141/169, 83.4%), stool tests were performed before optimizing the therapy to exclude infections. Over half of the physicians (101/169, 59.8%) optimized therapies based on clinical disease activity only (partial Mayo score ≥ 2 with rectal bleeding subscore ≥ 1 or stool frequency subscore ≥ 1) (Figure 1). Almost all participants (149/169, 88.2%) measured fecal calprotectin before optimizing the therapy. However, about half of the physicians (100/222, 45.1%) did not optimize the therapy in the event of an increase in fecal calprotectin alone (values > 250 µg/g). In this case, the timing of fecal calprotectin re-evaluation was heterogeneous: 3 months (33/96, 34.4%), 1 month (31, 32.3%), 2 months (15, 15.6%), 6 months (10, 10.4%), and ≤2 weeks (7, 7.3%). Most subjects (79/96, 82.3%) who did not optimize therapy based on fecal calprotectin alone repeated endoscopic procedures to assess disease activity. However, rectosigmoidoscopies/colonoscopies were performed after more than 3 months in a relevant percentage of cases: <6 months (4/17, 23.5%), <12 months (1, 5.9%), and >12 months (1, 5.9%). Similarly, CRP was frequently measured (146/169, 86.4%) before optimizing the therapy, but an increase in CRP (>5 mg/dL) alone was not sufficient to justify therapy optimization for approximately three-quarters of respondents (148/200, 74.0%). In these cases, a new CRP measurement was usually taken within 1 (58/148, 39.2%) or 3 (41, 27.7%) months. A relevant percentage of physicians (31/78, 39.8%) who requested ultrasound/radiologic tests to monitor disease activity did not change the therapy in the case of bowel wall thickness > 3 mm. Instead, two-thirds of respondents (116/169, 68.6%) performed an endoscopic evaluation before modifying the treatment. Nearly all physicians optimized therapies based on endoscopy findings (195/197, 99.0%). If the therapy was not escalated, a new endoscopic control was repeated after 4 months (2/2, 100%). Interestingly, in about half of the cases (64/145, 44.1%), the therapy was optimized if an endoscopic Mayo score of 1 was found. A similar percentage of physicians (61/145, 42.1%) increased the therapy in the case of histologic disease activity (e.g., a Nancy score ≥ 1 or the presence of neutrophils in the mucosa or in the lamina propria).

### 3.4. Follow-Up after Optimization

Clinical reassessment was generally performed 1 (53/169, 31.4%) or 3 months (49/169, 29.0%) after optimization (Table 2). Likewise, fecal calprotectin and CRP measurements were mostly taken after 1 (32/169, 18.9% and 54/169, 31.9%) or after 3 months (89/169, 52.7% and 65/169, 38.5%) of treatment, respectively. A colonoscopy/sigmoidoscopy was performed in only half of the cases (91/169, 53.8%) to monitor the response to therapy, mainly within 6 (36/91, 39.5%) or 3 months (23, 25.3%). Ultrasound/radiology was not performed in the majority of cases (95/166, 57.2%). On the other hand, if they were performed, the most frequent reassessments occurred 3 (28/71, 39.4%) and 6 months (19, 26.8%) after optimization. More than half of the physicians did not have a dedicated medical helpline (117/222, 52.7%) or email (129/222, 58.1%) for IBD patients experiencing a disease flare.

### 3.5. Treatment Decision Making

**Figure 2:**
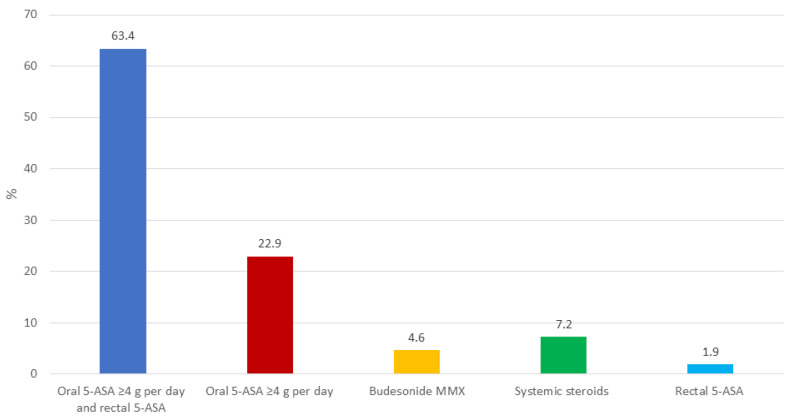
Preferred therapeutic option in a patient treated with 5-ASA (≤2 g per day) who experiences a relapse.

**Figure 3:**
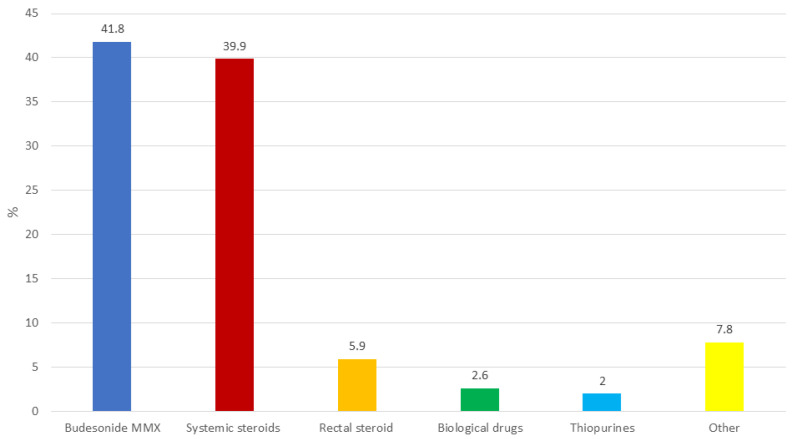
Preferred therapeutic option in a patient who is a non-responder to 5-ASA optimization.

In most cases, the therapeutic decision making regarding optimization took into consideration the severity of the disease (150/153, 98.0%) and the disease location (128/153, 83.7%). In a clinical scenario of a patient treated with 5-ASA (≤2 g per day) who experienced a relapse, the first choice was optimization with combined oral 5-ASA (≥4 g per day) and rectal 5-ASA (97/153, 63.4%), followed by treatment with oral 5-ASA (≥4 g per day) alone (35, 22.9%) (Figure 2). Only a small percentage of physicians preferred budesonide MMX (7, 4.6%) or systemic steroids (11, 7.2%). In the case of non-response to therapy, a new escalation was preferred within 2 (51/153, 33.3%) or 4 (50, 32.7%) weeks. In patients not responding to optimized 5-ASA, budesonide MMX (64/153, 41.8%) and systemic steroids (61, 39.9%) were the most frequently used drugs (Figure 3). A limited proportion of physicians preferred biologics (4/153, 2.6%). According to most participants (103/153, 67.3%), after adequate training, patients could optimize 5-ASA therapy autonomously based on clinical activity and fecal calprotectin values.

### 3.6. Therapy De-Escalation

In patients who achieved remission after optimization, de-escalation was recommended in two-thirds of cases (105/153, 68.6%). De-escalation generally occurred 2 (21/105, 20.0%) or 3 (39, 37.1%) months after achieving remission. In the case of relapse upon 5-ASA de-escalation (from ≥4 g/day to ≥2 g/day), it was common practice (133/153, 86.9%) to optimize the therapy again and maintain a stable 5-ASA dosage (≥4 g/day).

## 4. Discussion

To the best of our knowledge, this is the first survey specifically designed to investigate therapy optimization in patients with mild-to-moderate UC. In line with current guidelines, the combination of oral and rectal 5-ASA was the preferred first-line therapeutic option, while in the case of non-response to therapy, budesonide MMX and systemic steroids were used as the second-line treatments [11]. There is strong evidence supporting the use of combined oral and rectal 5-ASA in UC [19,20]. A randomized placebo-controlled clinical trial demonstrated that the combination of oral and rectal 5-ASA was superior to oral therapy alone in achieving clinical remission at week 8 (64% versus 43%, *p* = 0.03) [19]. A network meta-analysis of randomized clinical trials confirmed that the combination of rectal and oral 5-ASA was the best therapeutic option to achieve clinical and endoscopic remission in mild-to-moderate UC [20]. On the other hand, the high proportion of physicians who preferred systemic steroids after 5-ASA failure is surprising. Budesonide MMX has been associated with better outcomes compared to both 5-ASA and ileal-release budesonide [21,22]. Furthermore, it can be used as an add-on therapy to 5-ASA, allowing better disease control without affecting the safety profile [23]. To confirm its safety, a network meta-analysis including over 5000 patients with IBD showed that budesonide MMX was associated with significantly fewer adverse events compared with oral systemic corticosteroids [odds ratio (OR): 0.25, 95% confidence interval (CI): 0.13–0.49] [24]. In view of its efficacy and reliable safety, it is legitimate to hypothesize that budesonide MMX may be the first choice after 5-ASA failure. Systemic steroids, given the risk of side effects and steroid dependence, should be used in the case of budesonide MMX failure or in the case of severe disease. The survey also showed how CRP and fecal calprotectin are frequently used in daily clinical practice to monitor disease activity. However, although these tools are formally recognized as therapeutic targets in UC, a high proportion of physicians did not optimize therapies based on biochemical values alone [15]. This could be explained by the heterogeneity of fecal calprotectin and CRP measurements, which mase test results unreliable. To overcome this limitation, a recent expert consensus provided indications to standardize the measurement of fecal calprotectin, avoiding errors in the pre-analytical and analytical phases [25]. Importantly, only a few respondents monitored UC patients using radiological/ultrasound examinations. In addition, a considerable percentage of participants did not make treatment decisions based on imaging. Imaging, particularly intestinal ultrasound (IUS), has an increasing role in the management of UC. In fact, it predicts endoscopy and disease outcomes, therefore representing a rapid and non-invasive tool with which to assess disease activity and responses to therapy [26,27]. A recent prospective multicenter study demonstrated that IUS had better accuracy than endoscopy in predicting the risk of colectomy (AUROC 0.83, 95% CI: 0.75–0.92 vs. 0.71 95% CI: 0.62–0.80), thus supporting its widespread use. To date, there are no validated protocols for IUS, a defined training program is missing, and there are few centers with a high level of expertise [28]. Several initiatives of the International Bowel Ultrasound group (IBUS) are ongoing to address these issues and implement the adoption of IUS. On the other hand, the key role of endoscopy in the management of UC appears to be well established. Almost all physicians optimized therapy based on endoscopic data. Interestingly, a relevant percentage of them optimized therapy even in the case of mild endoscopic activity (an endoscopic Mayo score of 1). Growing evidence supports the use of this proactive approach. A prospective cohort study compared the risk of relapse in patients with endoscopic Mayo scores of 0 or 1 [29]. After 6 months of follow-up, there was a significantly higher number of relapses in patients with a Mayo score of 1 (36.6% vs. 9.4%, *p* < 0.001), and an endoscopic Mayo score of 1 was the only factor independently associated with the risk of recurrence (odds ratio 6.27, 95% confidence interval 2.73–14.40, *p* < 0.001). In addition, a meta-analysis confirmed that patients with an endoscopic Mayo score of 0 have a lower risk of clinical recurrence than those with an endoscopic Mayo score of 1 [30]. Of note, most physicians evaluated histologic activity (even in patients in remission) and histology was considered for therapeutic decisions in a considerable percentage of cases. A substantial proportion of histologic activity persists in patients with endoscopic remission [31]. Patients with endoscopic remission and concomitant histologic activity have an increased risk of experiencing a clinical recurrence of the disease [32]. The effort to achieve ever deeper remission has led to the identification of new composite endpoints, such as disease clearance [33]. This is defined as simultaneous clinical, endoscopic, and histologic remission [34]. Disease clearance is an achievable target with 5-ASA, and is associated with a reduced risk of hospitalization and surgery [35]. 

Treatment of mild-to-moderate UC is based on known drugs with proven efficacy. Their appropriate use plays a key role in controlling the disease. Likewise, the tight monitoring of patients can enable the early identification of disease relapses and the setting up of adequate treatment, therefore preventing complications. A recent decision-analytic Markov model compared a treat-to-target strategy with a symptom-based standard of care [36]. Interestingly, the treat-to-target approach was associated with a reduced risk of relapse and increased time spent in clinical and biochemical remission. An ongoing randomized clinical trial, the OPTIMISE study (NCT04340895), will provide further relevant evidence. The management of patients with mild-to-moderate UC based on clinical activity alone will be compared with an approach based on symptoms and fecal calprotectin. Our survey has several strengths, such as the high number of physicians involved and the number of nations represented, which support the reliability of the results obtained. However, there are some limitations of our survey which also need to be mentioned. First, not all the physicians answered all the questions. To overcome this limitation, the number of respondents for each question was provided. Second, the follow-up of patients could be affected by the waiting lists of individual hospitals, the availability of diagnostic procedures, and the expertise of each center. Moreover, physicians from different countries around the world were involved, suggesting that many factors beyond the doctors’ best knowledge (e.g., economic, social, cultural) may determine differences among the caregivers’ approach to IBD. 

In the near future, studies focused on the management of mild-to-moderate UC recurrences are warranted for advancing our understanding of the disease and improving patient care. Research in this area can help to establish standardized treatment protocols for relapses. This can ensure that all patients receive evidence-based care, leading to more consistent and effective management. In addition, understanding the factors that contribute to UC recurrences and responses to treatment can be associated with more personalized treatment plans by providing cost-effective and efficient ways to achieve disease control. 

## 5. Conclusions

Our survey provides a current snapshot of the management of patients with mild-to-moderate UC worldwide. The combination of oral and rectal 5-ASA should be the first therapeutic option, while budesonide MMX and systemic steroids should be considered in the case of non-response to 5-ASA. Although treatment targets are changing towards ever deeper remission, endoscopy is still the driver of therapeutic decisions in daily clinical practice. The use of non-invasive tools such as biomarkers and IUS, although steadily increasing, still elicits complaints from physicians.

## Figures and Tables

**Table 1:** Monitoring of patients with mild-to-moderate UC in clinical remission.

	n (%)
How often do you monitor patients in clinical remission? ➢ <3 months ➢ <6 months ➢ <9 months ➢ <12 months ➢ >12 months	27/222 (12.2%) 113 (50.9%) 36 (16.2%) 32 (14.4%) 14 (6.3%)
How often do you monitor fecal calprotectin levels in patients in clinical remission? ➢ <3 months ➢ <6 months ➢ <9 months ➢ <12 months ➢ >12 months How often do you monitor C-reactive protein levels in patients in clinical remission? ➢ <3 months ➢ <6 months ➢ <9 months ➢ <12 months ➢ >12 months How often do your patients in clinical remission undergo colonoscopy/rectosigmoidoscopy? ➢ Based on ECCO guidelines ➢ once a year ➢ every 2 years ➢ every 3 years ➢ every 5 years	19/222 (8.6%) 107 (48.2%) 35 (15.7%) 42 (18.9%) 19 (8.6%) 30/152 (19.7%) 77 (50.7%) 20 (13.1%) 17 (11.2%) 8 (5.3%) 100/197 (50.8%) 32 (16.2%) 43 (21.8%) 17 (8.6%) 5 (2.%)

n: number; ECCO: European Crohn’s and Colitis Organization.

**Table 2:** Monitoring of patients with mild-to-moderate UC after therapy optimization.

	n (%)
When do you assess the patient’s clinical activity after optimization? ➢ <2 weeks ➢ <1 month ➢ <2 months ➢ <3 months ➢ <6 months ➢ <12 months ➢ >12 months When do you assess fecal calprotectin levels after optimization? ➢ <2 weeks ➢ <1 month ➢ <2 months ➢ <3 months ➢ <6 months ➢ <12 months ➢ >12 months When do you assess C-reactive protein levels after optimization? ➢ <2 weeks ➢ <1 month ➢ <2 months ➢ <3 months ➢ <6 months ➢ <12 months ➢ >12 months When do you assess endoscopy after optimization? ➢ <1 month ➢ <2 months ➢ <3 months ➢ <6 months ➢ <12 months ➢ >12 months	22/169 (13.0%) 53 (31.4%) 26 (15.4%) 49 (29.0%) 17 (10.0%) 1 (0.6%) 1 (0.6%) 6/169 (3.5%) 32 (18.9%) 26 (15.4%) 89 (52.7%) 11 (6.5%) 3 (1.8%) 2 (1.2%) 15/169 (8.9%) 54 (31.9%) 21 (12.4%) 65 (38.5%) 10 (5.9%) 2 (1.2%) 2 (1.2%) 3/91 (3.3%) 3 (3.3%) 23 (25.3%) 36 (39.5%) 22 (24.2%) 4 (4.4%)

n: number.

## Data Availability

The data that support the findings of this study are available from the corresponding author upon reasonable request.

## Supplementary Materials

The following supporting information can be downloaded at: https://www.mdpi.com/article/10.3390/jcm13092510/s1.
