# Supplementary material for: Drug Optimization in Patients with Mild-to-Moderate Ulcerative Colitis: A Global Survey
Source: J Clin Med. 2024 Apr 24;13(9):2510. doi: 10.3390/jcm13092510 (PMC11084860; doi:10.3390/jcm13092510)
Supplement: Supplementary file 1 [file jcm-13-02510-s001.zip › jcm-2937399-SI.pdf]

Supplement data

Flowchart of the study

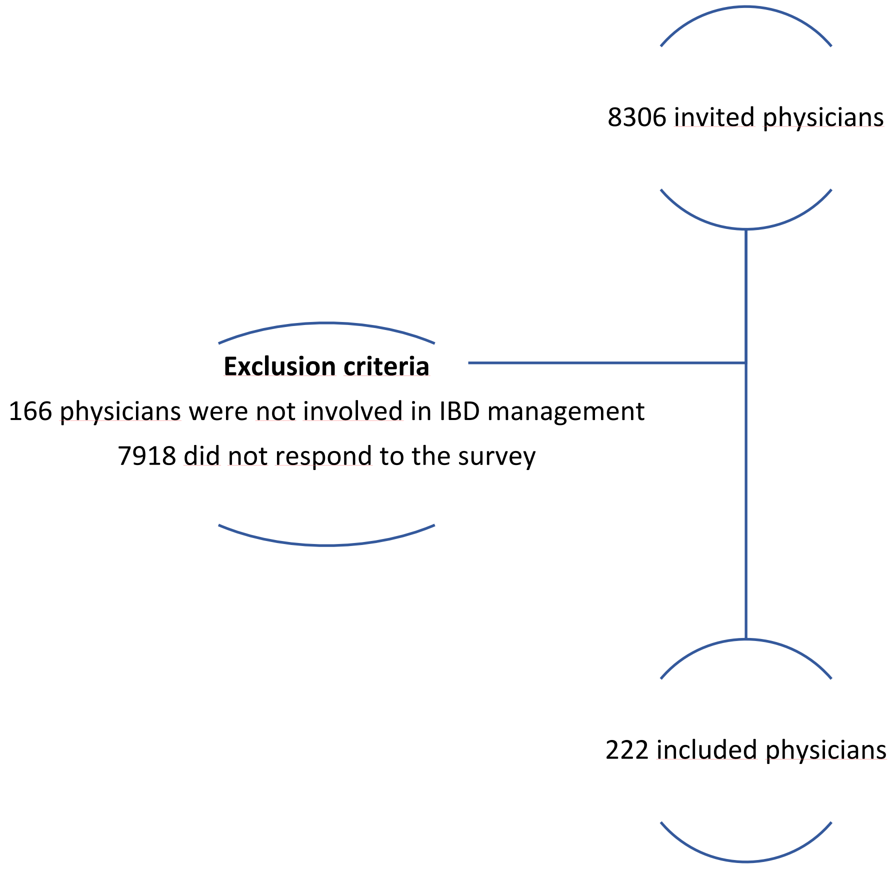

Number of participants by country

Countries	n (%)
Italy	30 (13.5%)
Brazil	15 (6.8%)
Greece	14 (6.3%)
Israel	10 (4.5%)
Poland	8 (3.6%)
Australia	6 (2.7%)
Serbia	6 (2.7%)
Argentina	5 (2.2%)
Croatia	5 (2.2%)
Hungary	5 (2.2%)
Ukraine	5 (2.2%)
United Kingdom	5 (2.2%)
Belgium	4 (1.8%)
India	4 (1.8%)
Lebanon	4 (1.8%)
Spain	4 (1.8%)
United Arab Emirates	4 (1.8%)
Vietnam	4 (1.8%)
Algeria	3 (1.3%)
Bangladesh	3 (1.3%)
Bulgaria	3 (1.3%)
Egypt	3 (1.3%)
Finland	3 (1.3%)
France	3 (1.3%)
Ireland	3 (1.3%)
Japan	3 (1.3%)
Jordan	3 (1.3%)
Mexico	3 (1.3%)
Switzerland	3 (1.3%)
Colombia	2 (0.9%)
Costa Rica	2 (0.9%)
Germany	2 (0.9%)
Indonesia	2 (0.9%)
Iran	2 (0.9%)
Kuwait	2 (0.9%)

Nigeria	2 (0.9%)
Peru	2 (0.9%)
Portugal	2 (0.9%)
Saudi Arabia	2 (0.9%)
Singapore	2 (0.9%)
South Africa	2 (0.9%)
Turkey	2 (0.9%)
Austria	1 (0.4%)
Canada	1 (0.4%)
Chile	1 (0.4%)
Czech Republic	1 (0.4%)
Denmark	1 (0.4%)
Honduras	1 (0.4%)
Hong Kong	1 (0.4%)
Iraq	1 (0.4%)
Kazakhstan	1 (0.4%)
Latvia	1 (0.4%)
Malaysia	1 (0.4%)
Mozambique	1 (0.4%)
Norway	1 (0.4%)
Pakistan	1 (0.4%)
Philippines	1 (0.4%)
Romania	1 (0.4%)
Slovakia	1 (0.4%)
Slovenia	1 (0.4%)
South Korea	1 (0.4%)
Taiwan	1 (0.4%)
Tunisia	1 (0.4%)
United States of America	1 (0.4%)
Uruguay	1 (0.4%)

Survey on mild to moderate UC optimization

1) Age in years:

2) Sex
   a) Male
   b) Female

3) What country do you work in?

4) What is your specialization?
   a) Gastroenterologist
   b) Internal doctor
   c) Surgeon
   d) General practitioner
   e) Other (Please specify)

5) How many years of experience do you have in the field of IBD?
   a) Less than 1 year
   b) Less than 5 years
   c) Less than 10 years
   d) More than 10 years

6) How many IBD patients do you visit per year?
   a) Less than 100
   b) Less than 500
   c) Less than 1000
   d) More than 1000

7) Which of the following therapies are available in your country (multiple choices are allowed)?
   a) 5-ASA
   b) Budesonide MMX
   c) Systemic steroids
   d) Immunosuppressants
   e) Biological drugs
   f) Small molecules

8) Does your hospital have a dedicated medical helpline for IBD patients experiencing a disease flare?
   a) Yes
   b) No

9) Does your hospital have a dedicated medical email for IBD patients experiencing a disease flare?
a) Yes
b) No

Patients in clinical remission of disease

10) How often do you monitor patients in clinical remission?
   a) < 3 months
   b) < 6 months
   c) < 9 months
   d) <12 months
   e) >12 months

11) Do you also visit patients in telemedicine?
   a) Yes
   b) No

12) Do you recommend using apps to monitor the clinical disease activity of your patients?
   a) Yes
   b) No

13) How often do you monitor fecal calprotectin levels in patients in clinical remission?
   a) < 3 months
   b) < 6 months
   c) < 9 months
   d) <12 months
   e) >12 months

14) Do you monitor fecal calprotectin levels through home testing?
   a) Yes
   b) No

15) Do you optimize therapy in case of increase of fecal calprotectin levels alone (>250µg/g)?
   a) Yes
   b) No

16) If you do not optimize therapy based on fecal calprotectin alone, how long after do you repeat fecal calprotectin measurement?
   a) ≤ 2 weeks
   b) 1 month
   c) 2 months
   d) 3 months
   e) 4 months
   f) 6 months

17) If you do not optimize therapy based on fecal calprotectin alone, do you perform colonoscopy/rectosigmoidoscopy to evaluate disease activity?
   a) Yes
   b) No

18) If you do not optimize therapy based on fecal calprotectin and perform colonoscopy/rectosigmoidoscopy, how long does it take before endoscopic examination is performed?
   a) < 4 weeks
   b) < 3 months
   c) < 6 months
   d) < 12 months
   e) > 12 months

19) How often do you monitor C-reactive protein levels in patients in clinical remission?
   a) < 3 months
   b) < 6 months
   c) < 9 months
   d) <12 months
   e) >12 months

20) Do you optimize therapy in case of increase of C-reactive-protein levels alone (>5 mg/dL)?
   a) Yes
   b) No
   c) Not applicable

21) If you do not optimize therapy based on C-reactive protein alone, how long after do you repeat C-reactive protein measurement?
   a) ≤ 2 weeks
   b) 1 month
   c) 2 months
   d) 3 months
   e) 4 months
   f) 6 months

22) How often do your patients in clinical remission undergo colonoscopy/rectosigmoidoscopy?
   a) Once a year
   b) Every 2 years
   c) Every 3 years
   d) Every 5 years
   e) Based on ECCO guidelines for colorectal cancer surveillance

23) Do you optimize therapy in case of endoscopic activity of disease (endoscopic Mayo score ≥2)?
   a) Yes
   b) No

24) If you do not optimize therapy based on endoscopic activity of disease, how long after do you repeat endoscopic assessment?
   a) 1 month
   b) 2 months
   c) 3 months
   d) 4 months

e) 6 months
f) 9 months
g) 12 months

25) Do you optimize therapy in case of mild endoscopic activity of disease (endoscopic Mayo score = 1)?
 a) Yes
 b) No

26) Do you take biopsies to monitor histological disease activity (even in patients in endoscopic remission)?
 a) Yes
 b) No

27) Do you optimize therapy in case of histologic activity of disease (e.g., Nancy score ≥1 or presence of neutrophils in the mucosa or in the lamina propria)?
 a) Yes
 b) No

28) Do you monitor disease activity through ultrasound/radiology?
 a) Yes
 b) No

29) If you monitor disease activity through ultrasound/radiology, how often do you request these exams?
 a) < 3 months
 b) < 6 months
 c) < 9 months
 d) <12 months
 e) >12 months

30) Do you optimize therapy in case of ultrasound/radiologic activity of disease (bowel wall thickness > 3mm)?
 a) Yes
 b) No
 c) Not applicable

Patients in clinical activity of disease

31) Before optimizing the therapy, do you always perform fecal tests to exclude infections (e.g. coproculture, parasites and clostridium difficile)?
 a) Yes
 b) No

32) Do you optimize therapy based on clinical disease activity only (partial Mayo score ≥2 with rectal bleeding subscore ≥1 or stool frequency subscore ≥1)?
 a) Yes

b) No

33) Do you measure faecal calprotectin levels before optimizing therapy?
a) Yes
b) No

34) Do you measure C-reactive-protein levels before optimizing therapy?
a) Yes
b) No

35) Do you perform colonoscopy/rectosigmoidoscopy before optimizing therapy?
c) Yes
d) No

36) Do you optimize therapy in case of ultasound/radiologic activity of disease (bowel wall thickness > 3mm)?
a) Yes
b) No
c) Not applicable

Monitoring after therapy optimization

37) When do you assess the patient's clinical activity after therapy optimization?
a) < 2 weeks
b) < 1month
c) < 2 months
d) < 3 months
e) < 6 months
f) < 12 months
g) > 12 months

38) When do you measure fecal calprotectin levels after therapy optimization?
a) < 2 weeks
b) < 1month
c) < 2 months
d) < 3 months
e) < 6 months
f) < 12 months
g) > 12 months

39) When do you measure C-reactive-protein levels after therapy optimization?
a) < 2 weeks
b) < 1month
c) < 2 months
d) < 3 months
e) < 6 months
f) < 12 months
g) > 12 months

40) Do you perform colonoscopy/rectosigmoidoscopy after therapy optimization?
   a) Yes
   b) No

41) If you answered yes to question 40, when do you perform colonoscopy/rectosigmoidoscopy after therapy optimization?
   a) < 1month
   b) < 2 months
   c) < 3 months
   d) < 6 months
   e) < 12 months
   f) > 12 months

42) Do you perform ultrasound/radiology after therapy optimization?
   a) Yes
   b) No

43) If you answered yes to question 42, when do you perform ultrasound/radiology after therapy optimization?
a) < 1month
b) < 2 months
c) < 3 months
d) < 6 months
e) < 12 months
f) > 12 months

Treatment

44) Does your treatment decision regarding optimization depend on severity of disease activity?
a) Yes
b) No

45) Does your treatment decision regarding optimization depend on location of disease activity (e.g., proctitis, left-sided colitis, or pancolitis)?
c) Yes
d) No

46) In a patient treated with 5-ASA (≤2 g per day) who experiences a relapse, what is your first-line option?
a) Oral 5-ASA (≥4 g per day)
b) Rectal 5-ASA
c) Oral 5-ASA (≥4 g per day) and rectal 5-ASA
d) Budesonide MMX
e) Systemic steroid

47) If a patient is a non-responder to 5-ASA optimization, when should medical therapy be escalated?

a) < 1 week
b) < 2 weeks
c) < 1 month
d) < 2 months
e) < 3 months
f) < 6 months

48) If a patient is a non-responder to 5-ASA optimization, what is your favourite option?
a) Budesonide MMX
b) Systemic steroid
c) Rectal steroid
d) Other (Please specify)

49) If a patient achieves disease remission after optimization, do you proceed with therapy de-escalation?
a) Yes
b) No

50) If a patient achieves disease remission after optimization, when should medical therapy be de-escalated?
a) < 2 weeks
b) < 1 month
c) < 2 months
d) < 3 months
e) < 6 months
f) < 12 months

51) Should a patient who relapses after de-escalation of 5-ASA therapy (from ≥4g/day to ≥2g/day) be re-escalated maintaining stable 5-ASA dosage (≥4g/day)?
a) Yes
b) No

52) Do you think that after adequate training, your patients could optimize 5-ASA therapy autonomously based on clinical activity and faecal calprotectin values?
   a) Yes
   b) No
   c) Do not know

Supplementary Table . CROSS checklist

Section/topic	Item	Item description	Reported on page #
**Title and abstract**			
Title and abstract	1a	State the word "survey" along with a commonly used term in title or abstract to introduce the study's design.	2,3
	1b	Provide an informative summary in the abstract, covering background, objectives, methods, findings/results, interpretation/discussion, and conclusions.	2
**Introduction**			
Background	2	Provide a background about the rationale of study, what has been previously done, and why this survey is needed.	3
Purpose/aim	3	Identify specific purposes, aims, goals, or objectives of the study.	3
**Methods**			
Study design	4	Specify the study design in the methods section with a commonly used term (e.g., cross-sectional or longitudinal).	4
Data collection methods	5a	Describe the questionnaire (e.g., number of sections, number of questions, number and names of instruments used).	4
	5b	Describe all questionnaire instruments that were used in the survey to measure particular concepts. Report target population, reported validity and reliability information, scoring/classification procedure, and reference links (if any).	4
	5c	Provide information on pretesting of the questionnaire, if performed (in the article or in an online supplement). Report the method of pretesting, number of times questionnaire was pre-tested, number and demographics of participants used for pretesting, and the level of similarity of demographics between pre-testing participants and sample population.	4
	5d	Questionnaire if possible, should be fully provided (in the article, or as appendices or as an online supplement).	Yes, in Data Suppl.
Sample characteristics	6a	Describe the study population (i.e., background, locations, eligibility criteria for participant inclusion in survey, exclusion criteria).	4,5
	6b	Describe the sampling techniques used (e.g., single stage or multistage sampling, simple random sampling, stratified sampling, cluster sampling, convenience sampling). Specify the locations of sample participants whenever clustered sampling was applied.	
	6c	Provide information on sample size, along with details of sample size calculation.	NA
	6d	Describe how representative the sample is of the study population (or target population if possible), particularly for population-based surveys.	NA
Survey	7a	Provide information on modes of questionnaire administration, including the type and number of contacts, the location where the survey was conducted (e.g., outpatient room	4

administration		or by use of online tools, such as SurveyMonkey).	
	7b	Provide information of survey's time frame, such as periods of recruitment, exposure, and follow-up days.	4
	7c	Provide information on the entry process:<br>–>For non-web-based surveys, provide approaches to minimize human error in data entry.<br>–>For web-based surveys, provide approaches to prevent "multiple participation" of participants.	4
Study preparation	8	Describe any preparation process before conducting the survey (e.g., interviewers' training process, advertising the survey).	NA
Ethical considerations	9a	Provide information on ethical approval for the survey if obtained, including informed consent, institutional review board [IRB] approval, Helsinki declaration, and good clinical practice [GCP] declaration (as appropriate).	NA
	9b	Provide information about survey anonymity and confidentiality and describe what mechanisms were used to protect unauthorized access.	4
Statistical analysis	10a	Describe statistical methods and analytical approach. Report the statistical software that was used for data analysis.	NA
	10b	Report any modification of variables used in the analysis, along with reference (if available).	NA
	10c	Report details about how missing data was handled. Include rate of missing items, missing data mechanism (i.e., missing completely at random [MCAR], missing at random [MAR] or missing not at random [MNAR]) and methods used to deal with missing data (e.g., multiple imputation).	4
	10d	State how non-response error was addressed.	NA
	10e	For longitudinal surveys, state how loss to follow-up was addressed.	NA
	10f	Indicate whether any methods such as weighting of items or propensity scores have been used to adjust for non-representativeness of the sample.	NA
	10g	Describe any sensitivity analysis conducted.	NA
**Results**			
Respondent characteristics	11a	Report numbers of individuals at each stage of the study. Consider using a flow diagram, if possible.	4,5
	11b	Provide reasons for non-participation at each stage, if possible.	NA
	11c	Report response rate, present the definition of response rate or the formula used to calculate response rate.	4,5
	11d	Provide information to define how unique visitors are determined. Report number of unique visitors along with relevant proportions (e.g., view proportion, participation proportion, completion proportion).	4,5

Descriptive results	12	Provide characteristics of study participants, as well as information on potential confounders and assessed outcomes.	4,5
	13a	Give unadjusted estimates and, if applicable, confounder-adjusted estimates along with 95% confidence intervals and p-values.	NA
Main findings	13b	For multivariable analysis, provide information on the model building process, model fit statistics, and model assumptions (as appropriate).	NA
	13c	Provide details about any sensitivity analysis performed. If there are considerable amount of missing data, report sensitivity analyses comparing the results of complete cases with that of the imputed dataset (if possible).	NA
**Discussion**			
Limitations	14	Discuss the limitations of the study, considering sources of potential biases and imprecisions, such as non-representativeness of sample, study design, important uncontrolled confounders.	10
Interpretations	15	Give a cautious overall interpretation of results, based on potential biases and imprecisions and suggest areas for future research.	8,9,10
Generalizability	16	Discuss the external validity of the results.	8,9,10
**Other sections**			
Role of funding source	17	State whether any funding organization has had any roles in the survey's design, implementation, and analysis.	16
Conflict of interest	18	Declare any potential conflict of interest.	17
Acknowledgements	19	Provide names of organizations/persons that are acknowledged along with their contribution to the research.	16
